# Assessment of Pediatrician Awareness and Implementation of the Addendum Guidelines for the Prevention of Peanut Allergy in the United States

**DOI:** 10.1001/jamanetworkopen.2020.10511

**Published:** 2020-07-15

**Authors:** Ruchi S. Gupta, Lucy A. Bilaver, Jacqueline L. Johnson, Jack W. Hu, Jialing Jiang, Alexandria Bozen, Jennifer Martin, Jamie Reese, Susan F. Cooper, Matthew M. Davis, Alkis Togias, Samuel J. Arbes

**Affiliations:** 1 Center for Food Allergy and Asthma Research, Institute for Public Health and Medicine, Northwestern University Feinberg School of Medicine, Chicago, Illinois; 2 Mary Ann & J. Milburn Smith Child Health Research, Outreach and Advocacy Center, Ann and Robert H. Lurie Children’s Hospital, Chicago, Illinois; 3 Rho Federal Systems Division Inc, Durham, North Carolina; 4 National Institute of Allergy and Infectious Diseases, Bethesda, Maryland

## Abstract

**Importance:**

The 2017 Addendum Guidelines for the Prevention of Peanut Allergy in the United States recommend that pediatricians assess infant peanut allergy risk and introduce peanut in the diet at age 4 to 6 months. Early introduction has the potential to prevent peanut allergy development.

**Objectives:**

To measure the rates of guideline awareness and implementation and to identify barriers to and factors associated with implementation among US pediatricians.

**Design, Setting, and Participants:**

This population-based study survey used a 29-item electronic survey instrument that was administered to pediatricians practicing across the United States from June 1, 2018, to December 1, 2018. Invitations to complete a survey were emailed to all pediatricians in the American Academy of Pediatrics vendor database. Eligible participants were nonretired US-based pediatricians providing general care to infants aged 12 months or younger.

**Main Outcomes and Measures:**

The primary outcome was the prevalence of guideline implementation, which was measured by 1 survey item about awareness followed by a second item about implementation. Secondary outcomes included identification of guidelines-focused services provided by pediatricians, knowledge of the guidelines (measured with 3 clinical scenarios), barriers to guideline implementation, need for training, and facilitators of guideline implementation.

**Results:**

A total of 1781 pediatricians were eligible to participate and completed the entire survey. Most respondents self-identified as white (1287 [72.5%]) and female (1210 [67.4%]) individuals. Overall, 1725 (93.4%; 95% CI, 92.2%-94.5%) pediatricians reported being aware of the guidelines. Of those pediatricians who had knowledge of the guidelines, 497 (28.9%; 95% CI, 26.8%-31.1%) reported full implementation and 1105 (64.3%; 95% CI, 62.0%-66.6%) reported partial implementation. Common barriers to implementation included parental concerns about allergic reactions (reported by 575 respondents [36.6%; 95% CI, 34.3%-39.1%]), uncertainty in understanding and correctly applying the guidelines (reported by 521 respondents [33.2%; 95% CI, 30.9%-35.6%]), and conducting in-office supervised feedings (reported by 509 respondents [32.4%; 95% CI, 30.1%-34.8%]). Many pediatricians (1175 [68.4%; 95% CI, 66.1%-70.5%]) reported a need for further training on the guidelines.

**Conclusions and Relevance:**

This survey found that most pediatrician respondents appeared to know of the 2017 guidelines, but less than one-third of respondents reported full implementation. Results of this study may inform future efforts to eliminate barriers to guideline implementation and adherence, thereby reducing the incidence of peanut allergy in infants.

## Key Points

**Question:**

What is the current rate of implementation of the 2017 Addendum Guidelines for the Prevention of Peanut Allergy in the United States, and what are the barriers to implementation among pediatricians who provide care for infants aged 12 months or younger?

**Findings:**

In this study survey of 1781 US pediatricians, 93% of respondents were aware of the guideline. Of those who had knowledge of the guidelines, 29% were fully implementing them and 64% were partially implementing them.

**Meaning:**

These findings suggest that understanding implementation barriers and needs among pediatricians is necessary to increase adherence and reduce peanut allergy incidence in infants.

## Teaser

This survey study describes the self-reported level of adherence, familiarity, and implementation needs associated with national peanut allergy prevention recommendations among pediatricians caring for infants.

## Introduction

Food allergy affects approximately 8% of children in the United States^1^ and is an increasing public health concern.^2^ The most common pediatric food allergy is peanut allergy, which has been reported in 2.2% of US children,^1^ is the least frequently outgrown among food allergies,^3^ and is often associated with severe reactions.^4^ In 2000, the American Academy of Pediatrics (AAP) released recommendations to delay the introduction of peanut to the diet until the child is aged 3 years.^5^ In 2008, the AAP published a clinical report demonstrating the lack of convincing evidence for delaying the introduction of peanut, but it did not provide further guidance.^6,7,8^

In 2015, the Learning Early About Peanut Allergy randomized clinical trial demonstrated that early introduction of peanut to infants between age 4 and 11 months who were at high-risk for developing peanut allergy resulted in a considerable reduction (81%) of peanut allergy prevalence by age 5 years.^9^ Based on these findings, the National Institute of Allergy and Infectious Diseases convened an expert panel, representing professional organizations, patient advocacy groups, and government agencies, to produce the Addendum Guidelines for the Prevention of Peanut Allergy in the United States.^10^ The guidelines were published in 2017 and included 3 recommendations. Recommendation 1 is that infants with severe eczema and/or egg allergy should undergo evaluation for allergic sensitization to peanut through specific IgE (sIgE) test and/or skin prick testing and, if necessary, an oral food challenge. Depending on the test results, peanut products should be introduced into the diet as early as 4 to 6 months of age (Figure). Recommendation 2 is that infants with mild to moderate eczema should begin peanut consumption around age 6 months. Recommendation 3 is that infants with no eczema or food allergy may consume peanut when age appropriate, in accordance with family preference and cultural practices.^10^

**Figure.**
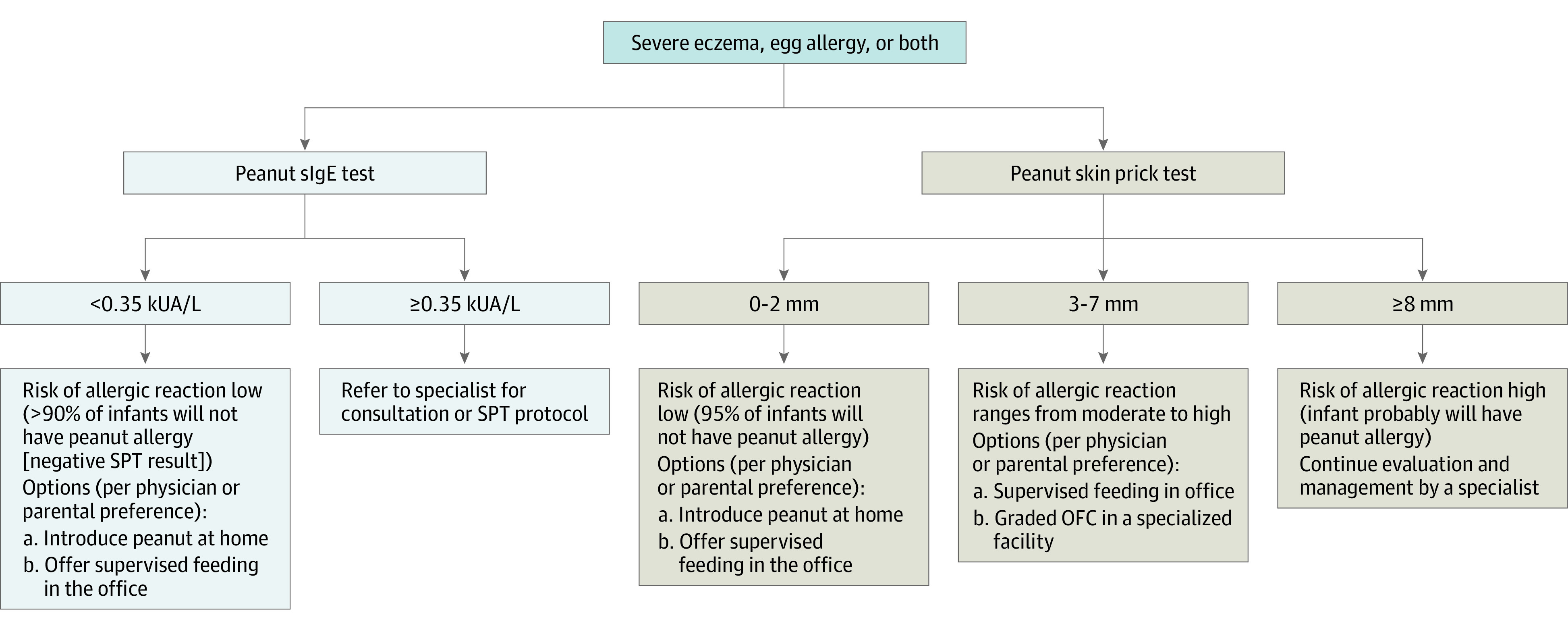
Recommendations for Evaluating Children With Severe Eczema and/or Egg Allergy Before Early Introduction of Peanut-Containing Products These recommendations are from the 2017 Addendum Guidelines for the Prevention of Peanut Allergy in the United States by the National Institute of Allergy and Infectious Diseases expert panel.^10^ OFC indicates oral food challenge; sIgE, specific IgE; SPT, skin prick testing.

Beginning with the 4 to 6–month well-child visits, pediatricians can have a vital role in guideline implementation and reduction in peanut allergy incidence. However, previous studies have suggested low rates of guideline implementation (use or application) and adherence (fidelity to recommendations) among pediatricians.^11,12,13^ Moreover, these studies^14^ included a small number of pediatricians in localized areas of the US and lacked an in-depth assessment of potential barriers to guideline implementation among pediatricians. We conducted the present study with the aim to measure the rates of guideline awareness and implementation as well as to identify barriers to and factors associated with implementation among US pediatricians.

## Methods

This population-based survey of US pediatricians was administered from June 1, 2018, to December 1, 2018. The study was reviewed and approved by the institutional review board of Ann & Robert H. Lurie Children’s Hospital of Chicago, which granted a waiver of signed informed consent because the research presented no more than minimal risk of harm to participants and involved no procedures for which written consent was required outside of the research context. As such, accessing the survey and answering survey questions implicitly indicated participant consent. We followed the American Association for Public Opinion Research (AAPOR) reporting guideline.

### Survey Development and Structure

Pediatricians throughout the United States were invited via email to participate in an online survey. The email invitation contained a link to the electronic survey. The survey instrument, which took less than 15 minutes to complete, included 29 fixed-response questions (eAppendix in the Supplement). Survey domains included demographic characteristics such as race/ethnicity, which survey participants self-selected. Additional domains included provision of guideline-related services and implementation, knowledge and training needs, barriers and facilitators of implementation, and participant and practice characteristics.

Categorical responses that included a free-text response (12 of 29 items had an “other” option) were evaluated by the study team (including members from the National Institute of Allergy and Infectious Diseases, Rho Federal Systems Division Inc, and Northwestern University Feinberg School of Medicine) and then were assigned to the listed categorical responses, if appropriate. If the free text could not be assigned to existing categories, it was added to the list of possible responses as “other.” Cognitive interviewing was used to pretest the initial survey instrument (9 pediatricians). We received feedback on the survey length, difficulty, questions, answer choices, wording, and order of questions. The study team reviewed the feedback and made revisions to the survey. The revised instrument was then uploaded on the online survey platform (Qualtrics) for a pilot test with 10 pediatricians.

### Study Design and Participants

The online survey was administered in 2 waves. Nonretired US pediatricians who provide general care to infants aged 12 months or younger were eligible to complete the survey. Eligibility was determined through the first 2 questions of the survey (eFigure in the Supplement). In wave 1, we used a vendor database obtained from the AAP to randomly select 7200 pediatricians with an email address, a US mailing address, and a listed practice type (excluding unclassified or other practice types). Because of the low survey response in wave 1, we conducted wave 2 with the remaining 37 446 pediatricians in the AAP vendor database who were not included in wave 1. Potential respondents in wave 2 and in the first 3 weeks of wave 1 were offered a $10 gift card as an incentive to complete the survey. Potential respondents in the last 2 weeks of wave 1 were offered a $50 gift card as an incentive, in our effort to increase the survey response rate.

### Outcome Measures

The primary outcome was the prevalence of guideline implementation, assessed by 1 item that asked about awareness of the guidelines, followed by a second item that asked about implementation (not using, using only parts of the guidelines, or using the guidelines as published and with few deviations) among those who were aware of the guidelines. Secondary outcomes were the guidelines-focused services provided by respondents, knowledge of the guidelines (measured with 3 clinical scenarios), barriers to implementation, need for training, and facilitators of implementation.

### Statistical Analysis

Waves 1 and 2 data were combined for analyses. Frequencies and percentages with 95% CIs were calculated for categorical responses. The CIs were calculated using the Clopper-Pearson method. Bivariate associations (practice location, practice region, academic affiliation, type of practice, percentage of patients with Medicaid, number of hours spent on pediatric care per week, mean number of years since medical school graduation, guidelines-focused services provided by pediatricians, and knowledge of the guidelines) with guideline implementation were tested with χ^2^ tests. Missing data attributed to incomplete surveys or imputation during text adjudication ranged from 0.2% to 5% across the survey items. Responders and nonresponders were compared by sex, number of years since medical school graduation, and practice region. The sensitivity of the primary outcome to survey wave (1 vs 2) and incentive level ($10 vs $50) was examined.

Two-sided hypothesis tests were used, with 2-sided *P* < .05 considered to be statistically significant. All statistical calculations were performed with SAS, version 9.4 (SAS Institute Inc). Data were analyzed from December 15, 2018, to October 31, 2019.

## Results

### Participants

Of the 41 048 email invitations sent, 2 135 pediatricians (5.2%) responded to the first question (What is your primary medical specialty?). After exclusion of ineligible pediatricians who did not primarily specialize in pediatrics and/or did not provide general pediatric care to infants aged 12 months or younger, a total of 1868 pediatricians were eligible to participate. Among the 1868 pediatricians, 1781 (95.3%) completed the full survey (eFigure in the Supplement).

Most participants self-identified as white (1287 [72.5%]) and female (1210 [67.4%]) individuals. More than half of the respondents (972 [54.4%]) practiced in a suburban location, whereas 196 (11.0%) practiced in a rural location. Practicing without an academic affiliation (1222 [68.3%]) and in a private group practice (799 [44.9%]) were most common. The demographic characteristics (sex and race/ethnicity) of the study sample were similar to statistics reported by the AAP, with the exception of practicing in a private group practice (44.9% in the present survey vs 33.3% in the AAP report^15^) and in an urban location (34.7% in the present survey vs 49.5% in the AAP report^15^). Participant demographic characteristics and a comparison with the AAP membership report are presented in Table 1. Demographic characteristics did not vary by wave, and no differences in sex, number of years since medical school graduation, and practice region were observed between survey participants and nonparticipants.

**Table 1.** Demographic Distribution of Pediatrician Respondents and Practice Information

Characteristic	Present survey		AAP report, %^b^^,^^c^
	No.^a^	% (95% CI)^b^	
Race/ethnicity			
Hispanic or Latino	117	6.5 (5.4-7.7)	6.2
Not Hispanic or Latino	1679	93.5 (92.2-94.6)	NR
White	1287	72.5 (70.4-74.6)	73
Asian	375	21.1 (19.2-23.1)	16.9
Black or African American	96	5.4 (4.4-6.6)	4.9
Native Hawaiian or other Pacific Islander	13	0.7 (0.4-1.2)	NR
American Indian or Alaskan native	6	0.3 (0.1-0.7)	NR
Other	22	1.2 (0.8-1.9)	1.9
Sex			
Female	1210	67.4 (65.2-69.6)	63.7
Male	584	32.6 (30.4-34.8)	36.3
Practice location			
Suburban	972	54.4 (52.0-56.7)	39.8
Urban	620	34.7 (32.5-36.9)	49.5
Rural	196	11.0 (9.6-12.5)	10.6
Practice region			
Midwest	345	19.4 (17.6-21.3)	NR
Northeast	467	26.2 (24.2-28.3)	NR
South	597	33.5 (31.3-35.7)	NR
West	373	20.9 (19.1-22.9)	NR
Academic affiliation			
Yes	566	31.7 (29.5-33.9)	NR
No	1222	68.3 (66.1-70.5)	NR
Type of practice			
Private: group practice	799	44.9 (42.5-47.2)	33.3
Hospital practice or clinic	263	14.8 (13.2-16.5)	14.6
Academic medical center practice or clinic	247	13.9 (12.3-15.6)	15.3
Private: solo practice	199	11.2 (9.7-12.7)	10.5
Community clinic or community health center	167	9.4 (8.1-10.8)	3.1
Managed care center/HMO	76	4.3 (3.4-5.3)	2.4
Military or US government	24	0.3 (0.1-0.7)	NR
Other	6	1.3 (0.9-2.0)	6.2
Patients with Medicaid, %			
0-25	686	38.5 (36.3-40.8)	NR
26-50	480	27.0 (24.9-29.1)	NR
51-75	363	20.4 (18.5-22.3)	NR
76-100	252	14.1 (12.6-15.9)	NR
Hours spent on pediatric care			
No. of h/wk, mean (SD)	1791	36.9 (13.2)	32.8
Full-time: ≥40 h	966	53.9 (51.6-56.3)	NR
Part-time: ≤39 h	825	46.1 (43.7-48.4)	26
Mean No. of years since medical school graduation, y			
0-10	231	12.9 (11.4-14.5)	NR
11-20	463	25.8 (23.8-27.9)	NR
21-30	550	30.7 (28.5-32.9)	NR
≥31	549	30.6 (28.5-32.8)	NR

Abbreviations: AAP, American Academy of Pediatrics; HMO, health maintenance organization; NR, not reported.

^a^ Number of observations available for each variable differs because of missing data.

^b^ Percentages may not add to 100 because of rounding.

^c^ In 2016, the AAP had approximately 67 000 members.^15^

### Services Provided by Pediatricians

Most responding pediatricians offered counseling to parents on peanut allergy prevention (n = 1700 [91.6%; 95% CI, 90.3%-92.9%]) and referrals to allergists (n = 1672 [90.1%; 95% CI, 88.7%-91.5%]). Nearly half of respondents (n = 904 [48.7%; 95% CI, 46.4%-51.0%]) performed a peanut sIgE test. Less than 10% of respondents reported providing other guidelines-focused services, including in-office supervised feedings (n = 161 [8.7%; 95% CI, 7.4%-10.1%]), graded oral food challenge (n = 120 [6.5%; 95% CI, 5.4%-7.7%]), and peanut-specific skin prick testing (n = 75 [4.0%; 95% CI, 3.2%-5.0%]).

### Implementation, Knowledge, and Training Needs

Most participants (n = 1725 [93.4%; 95% CI, 92.2%-94.5%]) were aware of the guidelines. Sources of information on the guidelines included medical journals, publications from professional organizations, continuing medical education courses, and word of mouth from colleagues (Table 2). Nearly all respondents who were aware of the guidelines reported being very familiar (n = 687 [39.9%; 95% CI, 37.6%-42.3%]) or somewhat familiar (n = 991 [57.6%; 95% CI, 55.2%-59.9%]) with the content. Among those with knowledge of the guidelines, 497 (28.9%; 95% CI, 26.8%-31.1%) were fully implementing the guidelines, whereas 1105 (64.3%; 95% CI, 62.0%-66.6%) reported partial implementation (ie, “using parts of the 2017 guidelines but not all of it”) (Table 2). Neither knowledge nor implementation substantially differed by wave or incentive level (eTable 1 in the Supplement).

**Table 2.** Guideline Implementation, Knowledge, and Training Needs

Variable	No. (%) [95% CI]^a^
Knowledge of the guidelines^b^	
Very familiar	687 (39.9) [37.6-42.3]
Somewhat familiar	991 (57.6) [55.2-59.9]
Not familiar	43 (2.5) [1.8-3.4]
Implementation of the guidelines^c^	
Full	497 (28.9) [26.8-31.1]
Partial	1105 (64.3) [62.0-66.6]
None	116 (6.8) [5.6-8.0]
Source of information on the guidelines^c^	
Medical journals	1240 (72.1) [69.9-74.2]
Articles or notices from professional organizations	1111 (64.6) [62.3-66.9]
Continuing medical education courses	707 (41.1) [38.8-43.5]
Word of mouth from medical colleagues	697 (40.5) [38.2-42.9]
News stories	511 (29.7) [27.6-31.9]
Expert lectures or grand rounds	328 (19.1) [17.2-21.0]
Local, state, national, or international medical meetings	284 (16.5) [14.8-18.4]
Online social media	114 (6.6) [5.5-7.9]
Advocacy or health care organizations	109 (6.3) [5.2-7.6]
My residency or fellowship	94 (5.5) [4.4-6.6]
Online tutorials or courses	93 (5.4) [4.4-6.6]
In-service training within my practice	71 (4.1) [3.2-5.2]
Other	20 (1.2) [0.7-1.8]
Need for guideline training	
Yes	1175 (68.4) [66.1-70.5]
No	544 (31.6) [29.5-33.9]

^a^ Percentages may not add to 100 because of rounding.

^b^ The term *guidelines* refers to the 2017 Addendum Guidelines for the Prevention of Peanut Allergy in the United States by the National Institute of Allergy and Infectious Diseases expert panel.^10^

^c^ Implementation of the guidelines and sources of information on the guidelines were reported by pediatricians who were aware of the guidelines.

When asked to specify peanut introduction recommendations for patients of varying (high, moderate, and low) peanut allergy risk categories, 728 participants (40.6%; 95% CI, 38.2%-42.9%) provided correct answers to all 3 scenarios. Most respondents (n = 1531 [84.4%; 95% CI, 82.6%-86.0%]) correctly indicated that, when presented with a 6-month-old infant without eczema or any food allergies (scenario 1), they would recommend the introduction of peanut-containing food in accordance with family preferences and cultural practices (Table 3). For infants with mild to moderate eczema (scenario 2), 987 respondents (54.7%; 95% CI, 52.4%-57.0%) indicated they would recommend introducing peanut products in accordance with the guidelines. For infants with severe eczema and/or egg allergy (scenario 3), 1079 participants (59.8%; 95% CI, 57.5%-62.1%) indicated they would refer the infant to an allergist for consultation and 341 participants (18.9%; 95% CI, 17.1%-20.8%) would order a peanut sIgE test; both approaches are consistent with the guidelines. However, 375 participants (20.8%; 95% CI, 18.9%-22.7%) indicated they would pursue approaches not consistent with the guidelines. Overall, 1175 pediatricians (68.4%; 95% CI, 66.1%-70.5%) reported a need for further guideline training.

**Table 3.** Survey Responses to 3 Clinical Scenarios Regarding Peanut Allergy Prevention

Survey item	No. (%) [95% CI] of responses to select answer options^a^
**Scenario 1: For an infant aged 6 mos who does NOT have eczema or any food allergies, what would you typically do next with respect to peanut allergy prevention? (Select only one)**	
Recommend the introduction of peanut-containing food, in accordance with family preferences and cultural practices	1531 (84.4) [82.6-86.0]
I would not take any additional steps with respect to peanut allergy prevention	100 (5.5) [4.5-6.7]
Recommend avoidance of peanut-containing foods	75 (4.1) [3.3-5.2]
Refer to an allergist for consultation and testing	60 (3.3) [2.5-4.2]
Offer an in-office feeding of a peanut-containing food	24 (1.3) [0.8-2.0]
Order a peanut-specific IgE test	14 (0.8) [0.4-1.3]
Conduct peanut-specific skin prick testing in my office	6 (0.3) [0.1-0.7]
Other	4 (0.2) [0.1-0.6]
**Scenario 2: For an infant aged 6 mos who has mild-to-moderate eczema, what would you typically do next with respect to peanut allergy prevention? (Select only one)**	
Recommend the introduction of peanut-containing food	987 (54.7) [52.4-57.0]
Refer to an allergist for consultation and testing	238 (13.2) [11.7-14.8]
Order a peanut-specific IgE test	228 (12.6) [11.1-14.3]
Recommend avoidance of peanut-containing food	126 (7.0) [5.9-8.3]
Offer an in-office feeding of peanut-containing food	103 (5.7) [4.7-6.9]
I would not take any additional steps with respect to peanut allergy prevention	96 (5.3) [4.3-6.5]
Other	20 (1.1) [0.7-1.7]
Conduct peanut-specific skin prick testing in my office	6 (0.3) [0.1-0.7]
**Scenario 3: For an infant aged 6 mos who has severe eczema and/or egg allergy, what would you typically do next with respect to peanut allergy prevention? (Select only one)**	
Refer to an allergist for consultation and testing	1079 (59.8) [57.5-62.1]
Order a peanut-specific IgE test	341 (18.9) [17.1-20.8]
Recommend the introduction of peanut-containing food	157 (8.7) [7.4-10.1]
Recommend avoidance of peanut-containing food	124 (6.9) [5.8-8.1]
Offer an in-office feeding of peanut-containing food	57 (3.2) [2.4-4.1]
I would not take any additional steps with respect to peanut allergy prevention	33 (1.8) [1.3-2.6]
Conduct peanut-specific skin prick testing in my office	8 (0.4) [0.2-0.9]
Other	4 (0.2) [0.1-0.6]

^a^ Percentages may not add to 100 because of rounding.

### Implementation Barriers and Facilitators 

Among respondents who were fully or partially implementing the guidelines, the barriers were categorized into 3 types (Table 4). Of the pediatrician- and practice-related barriers to implementation, the most frequently identified were conducting in-office supervised feedings of peanut (n = 509 [32.4%; 95% CI, 30.1%-34.8%]) and lack of clinic time (n = 450 [28.7%; 95% CI, 26.5%-31.0%]). Participants also reported that conducting peanut-specific IgE antibody testing was a barrier to implementation (n = 231 [14.7%; 95% CI, 13.0%-16.6%]). Of the familiarity or acceptance barriers, more than one-third of respondents reported that understanding and correctly applying the guidelines were a barrier (n = 521 [33.2%; 95% CI, 30.9%-35.6%]). Among the parental concerns as a barrier, 575 participants (36.6%; 95% CI, 34.3%-39.1%) reported parental concerns about allergic reactions.

**Table 4.** Implementation Barriers and Preferred Practice Aids or Office Materials^a^

Variable	No. (%) [95% CI]
Barriers to and concerns about implementing the guidelines	
Pediatrician- and practice-related issues	
Conducting an in-office supervised feeding of peanut	509 (32.4) [30.1-34.8]
Lack of clinic time	450 (28.7) [26.5-31.0]
Conducting peanut-specific IgE antibody testing	231 (14.7) [13.0-16.6)
Pediatrician concerns about allergic reactions	215 (13.7) [12.0-15.5]
Legal liability	166 (10.6) [9.1-12.2]
Access to an allergist for referrals	146 (9.3) [7.9-10.9]
Insufficient insurance coverage or reimbursement	130 (8.3) [7.0-9.8]
Familiarity or acceptance	
Understanding and correctly applying the guidelines	521 (33.2) [30.9-35.6]
Newness of the guidelines	400 (25.5) [23.4-27.7]
Pediatrician disagrees with part or all of the guidelines	42 (2.7) [1.9-3.6]
Parental concerns	
Parental concerns about allergic reactions	575 (36.6) [34.3-39.1]
Parental concerns about blood draws	315 (20.1) [18.1-22.1]
Parents who are not interested	226 (14.4) [12.7-16.2]
Preferred practice aids to assist implementation	
An online tutorial on guideline implementation	827 (52.8) [50.3-55.3]
Prompts in the electronic medical health record	500 (31.9) [29.6-34.3]
A printed or electronic handout to guide clinical assessments and recommendations	944 (60.3) [57.8-62.7]
A printed or electronic script for explaining the guidelines to parents	860 (54.9) [52.4-57.4]
A printed or electronic handout to guide in-office supervised feeding	524 (33.5) [31.1-35.9]
Other	1 (0.1) [0.0-0.4]
I am not interested in practice aids	56 (3.6) [2.7-4.6]
Preferred office materials to assist implementation	
A waiting room poster about peanut allergy prevention	733 (46.9) [44.4-49.4]
A paper or electronic handout explaining the guidelines	957 (61.2) [58.8-63.7]
A paper or electronic handout that provides answer to frequently asked questions	1122 (71.8) [69.5-74.0]
A paper or electronic handout on the feeding of peanut-containing foods at home	1143 (73.1) [70.9-75.3]
Other	11 (0.7) [0.4-1.3]
I am not interested in any office material for parents	59 (3.8) [2.9-4.8]

^a^ The term *guidelines* refer to the 2017 Addendum Guidelines for the Prevention of Peanut Allergy in the United States by the National Institute of Allergy and Infectious Diseases expert panel.^10^

Responding pediatricians indicated the need for a variety of practice aids and office materials to facilitate implementation of the guidelines (Table 4). The most preferred office materials for parents were handouts on feeding their infants peanut-containing foods at home (n = 1143 [73.1%; 95% CI, 70.9%-75.3%]). Frequently-asked-questions handouts (n = 1122 [71.8%; 95% CI, 69.5%-74.0%]), and handouts explaining the guidelines for parents (n = 957 [61.2%; 95% CI, 58.8%-63.7%]) were also preferred. Preferred practice aids that would be helpful according to pediatricians included printed or electronic handouts to guide clinical assessments and recommendations (n = 944 [60.3%; 95% CI, 57.8%-62.7%]), a printed or electronic script for explaining the guidelines to parents (n = 860 [54.9%; 95% CI, 52.4%-57.4%]), an online tutorial on guidelines implementation (n = 827 [52.8%; 95% CI, 50.3%-55.3%]), and prompts in the electronic medical health record (n = 500 [31.9%; 95% CI, 29.6%-34.3%]).

### Association With Pediatrician and Practice Characteristics

eTable 2 in the Supplement presents associations between practice characteristics and selected outcomes (guideline implementation, need for training, and barriers to implementation). Specifically, having more patients with Medicaid and practicing in a rural location were associated with higher rates of not implementing the guidelines, with 24 respondents serving 76% to 100% patients with Medicaid reporting that they did not implement compared with 42 respondents serving 0% to 25% patients with Medicaid (n = 24 [10.3%; 95% CI, 6.7%-14.9%] vs n = 42 [6.3%; 95% CI, 4.6%-8.5%]; *P* = .03). Similarly, more participants practicing in rural locations did not implement the guidelines compared with those in suburban and urban locations (n = 15 [8.2%; 95% CI, 4.7%-13.2%] vs n = 59 [6.4%; 95% CI, 4.9%-8.2%] and n = 42 [7.4%; 95% CI, 5.4%-9.9%]; *P* = .03). In addition, more participants with the highest Medicaid patient population of 76% to 100% reported needing training on the guidelines compared with participants with the lowest Medicaid patient population of 0% to 25% (n = 177 [76.0%; 95% CI, 70.0%-81.3%] vs n = 415 [62.7%; 95% CI, 58.9%-66.4%]; *P* < .001). Need for training was also reported to be higher for participants practicing in a rural vs an urban location (n = 124 [68.1%; 95% CI, 60.8%-74.8%] vs n = 414 [72.9%; 95% CI, 69.0%-76.5%]; *P* < .02). Access to allergists for referrals was more commonly reported as a barrier by participants with the highest compared with the lowest Medicaid population (n = 33 [15.8%; 95% CI, 11.1%-21.5%] vs n = 36 [5.8%; 95% CI, 4.1%-7.9%]; *P* < .001) and by participants practicing in a rural area vs suburban and urban areas (n = 28 [16.8%; 95% CI, 11.4%-23.3%] vs n = 61 [7.0%; 95% CI, 5.4%-9.0%] and n = 57 [10.8%; 95% CI, 8.3%-13.8%]; *P* < .001).

## Discussion

To our knowledge, this study is the first population-based survey of a large, nationwide sample of US pediatricians that characterizes the current practices and barriers associated with the 2017 Addendum Guidelines for the Prevention of Peanut Allergy in the United States. This survey was conducted 1.5 years after the publication of the guidelines. Although most pediatrician respondents indicated guideline awareness, only 28.9% reported implementing the published guidelines fully or with a few deviations. When 3 clinical scenarios were presented that would demonstrate correct implementation, only 40.6% of participants provided guideline-compatible answers for infants in all 3 risk categories (high, moderate, and low risk). Common barriers to guideline implementation included practice issues such as lack of clinic time, conducting in-office supervised feeding of peanut, and conducting a peanut sIgE test; pediatrician issues, including knowledge of or comfort with the guidelines and concerns about the newness of the guidelines; and parental issues such as fear of allergic reactions. Nearly 70% (68.4%) of pediatricians reported needing additional guideline training.

A high level of awareness of the guidelines with low implementation among pediatricians was observed. However, the degree of implementation appeared to be higher than in previous reports that assessed smaller, local samples of pediatricians. In the Hoffman et al^12^ survey of pediatricians at an academic medical center, 11% of respondents achieved high rates of adherence; however, this study was conducted soon after the publication of the guidelines. More recent studies involving medical record review of infants at the 4 to 6–month well-child visits have also suggested low rates of guideline adherence.^11,16^ In a review of 312 infant well-child visits in a Chicago clinic, pediatricians were fully adherent to the guidelines for only 14% of infants, regardless of risk level.^11^ Tapke et al^16^ indicated that early peanut introduction was discussed in 3.3% of the 4 to 6–month well-child visits of infants with high risk. These data raise the possibility that despite their knowledge of and willingness to adhere to the guidelines, pediatricians may encounter practical obstacles that decrease implementation rates. These findings echo the experience of physicians after publication of asthma guidelines, whereby implementation lagged behind awareness.^17^

When all pediatricians were asked about the first clinical scenario, 84.4% indicated they would recommend peanut products to infants at low risk and those without eczema and/or food allergies. This rate is a substantial achievement given that the guidelines had only been published for 1.5 years before the administration of this survey and is a reversal from the response to the 2000 AAP recommendations for caregivers to wait until their child is aged 3 years to initiate consumption of peanut products. It is likely that up to one-quarter of peanut allergy cases are derived from a low-risk population,^18^ and the finding that most pediatrician respondents would now recommend peanut introduction to such infants may alter the incidence of peanut allergy.

Implementation of the guidelines for infants at moderate risk for peanut allergy (mild to moderate eczema) was a challenge for pediatricians. In this second scenario, only half of pediatricians (54.7%) reported advising peanut product introduction, in accordance with guidelines. Given the absence of practical matters that could be considered barriers to implementation, it is important to better understand whether this finding represents confusion in ascertaining eczema severity or fear of reactions by the pediatrician or caregivers. A deeper understanding of the issues may provide an opportunity for education and intervention in this area.

In the third scenario referring to high-risk infants (severe eczema and/or egg allergy), more than half of pediatricians (59.8%) responded that they would refer to an allergist. However, 18.9% of respondents indicated they would order a peanut sIgE test, which is a measure of allergic sensitization and not clinical food allergy. The guidelines recommend that pediatricians use the sIgE test as an alternative to allergist referral to minimize the delay in peanut introduction for infants whose sIgE test results may be negative.^10^ Because a negative result has an extremely low risk of reaction (more than 90% negative predictive value),^19^ pediatricians can safely recommend peanut introduction, thus reducing the need for allergy referrals and avoiding possible bottlenecks.^20^ Previous research has indicated that both urban and rural areas face challenges in accessing specialty health care services,^21^ therefore pediatricians who screen for peanut allergy could help provide an allergy assessment for more infants. If the sIgE test result is positive, an infant may or may not be allergic to peanut and should be referred to an allergist for further assessment. It is important to understand the reasons pediatricians do not use an sIgE test because this area may be another potential opportunity for education and training.

When asked what they would recommend for infants with severe eczema and/or egg allergy, 20.8% of pediatricians reported offering advice that was inconsistent with the guidelines, including 8.7% of pediatricians who would recommend peanut introduction without referring a patient to an allergist or performing an sIgE test or skin prick testing. Risk assessment of infants for peanut allergy is recommended only in the US, whereas other countries recommend early introduction of peanut for all infants regardless of risk.^22^ Although, in general, infants exhibit mild symptoms of the skin and gastrointestinal system and fewer respiratory and cardiovascular symptoms compared with older children,^23^ the overall evidence is limited. Furthermore, the National Institute of Allergy and Infectious Diseases expert panel that published the guidelines chose a more conservative approach to avoid jeopardizing the transition to introduction of peanut-containing food, which has the potential to substantially decrease the prevalence of peanut allergy.

The most common barrier to guideline implementation, reported by 28.7% of pediatricians, was lack of clinic time. This finding is consistent with results of previous research on other guidelines, which has suggested that barriers to implementation include physician knowledge, attitude, and clinical factors such as lack of time or resources.^17,24^ Lack of time is not surprising given the many child health and development priorities discussed during visits.^25^ Adding guideline-specified practices around peanut introduction to a well-child visit is challenging. Availability of electronic or paper educational materials for parents may partially alleviate this problem. According to 32.4% of pediatrician respondents, in-office feedings were a barrier to guideline implementation: less than 10% reported conducting in-office feedings. Considering the time constraints in a pediatric practice, this limitation is understandable. However, if the peanut sIgE test result is negative, the guidelines support the introduction of peanut at home for high-risk infants. Therefore, pediatricians have little need to conduct in-office feedings. Conducting a peanut sIgE test was reported as a barrier by 14.7% of pediatricians, and only 48.7% of respondents reported providing this guideline-focused service. In a previous survey study on physician knowledge of food allergy, 47% of primary care physicians, pediatricians, and family physicians did not feel comfortable with ordering an sIgE test for food allergy, and only 29% reported being comfortable with interpreting diagnostic food allergy tests.^26^ This finding supports the notion that increasing the knowledge and comfort level of pediatricians under the conditions specified by the guidelines could prove valuable in the overall promotion of early peanut introduction. In the present survey, 36.6% of pediatricians identified parental concerns about allergic reactions as a barrier to guideline implementation. A parallel survey of allergists also identified these parental concerns as a barrier.^27^ Previous studies indicated that parents or caregivers were concerned about the early introduction of allergenic foods.^28^ In a recent report, only 31% of parents or caregivers were willing to introduce peanut-containing food at or before age 6 months, as recommended in the guidelines.^28^ In a review of allergy clinic medical records, almost 40% of high-risk infants referred for evaluation never returned for their oral food challenge.^29^ These past observations further emphasize the need for a campaign that will familiarize parents with the guidelines, an approach that has been successfully implemented in Australia.^30^ Overall, the barriers that US pediatricians encounter in guideline implementation need to be addressed and warrant further attention.

We found few differences in implementation according to practice types or pediatrician demographic characteristics. The main differences were seen for pediatricians serving a larger Medicaid population and those practicing in rural locations. Both groups were statistically significantly less likely to implement the guidelines and more likely to report a need for training. These pediatricians were also more likely to report access to allergist referrals as a barrier to implementation. Previous studies have suggested the difficulty of obtaining new patient specialty visits for patients with Medicaid and the often long wait times.^31,32^ Providing additional resources to pediatricians in rural environments or those who serve the Medicaid population is a crucial priority. In this survey, 33.2% of respondents indicated that understanding and correctly applying the guidelines was a common barrier to implementation, and 68.4% reported a need for training, confirming the need for medical education in specific areas. Pediatricians identified several practice aids or office materials to assist in guideline implementation, including tutorials, prompts in the electronic health record, and handouts or scripts. Successful interventions for increasing clinical implementation in pediatric primary care have involved clinical decision support tools.^33,34,35,36,37,38,39,40,41^ Further research into forms of training and types of practice aids is necessary to increase implementation. Future revisions to the guidelines should also be considered to improve their penetrability.

### Limitations

This study has some limitations. A major limitation is the low survey response rate of 5.2%, which raises concerns about the generalizability of the study findings. Although the characteristics of the pediatricians who participated in the survey are similar to those of the overall membership of the AAP, the results may suffer from participation bias. Of particular concern is that pediatricians who were more knowledgeable of the guidelines might have been more likely to complete the survey. However, the levels of guideline awareness and adherence were comparable to those reported by other, smaller studies.^11,12^ Another limitation is that the findings are not representative of pediatrician guideline implementation in other countries such as the United Kingdom and Australia, where universal peanut allergy risk screening for infants is not recommended. However, the present study focused on the implementation of the US guidelines, which differ from those in other countries.

## Conclusions

Pediatrician awareness and partial implementation of the 2017 Addendum Guidelines for the Prevention of Peanut Allergy in the United States appeared to be high; however, full implementation of the guidelines seemed low. Although the guidelines had been published for only 1.5 years when this survey was conducted, most of the pediatrician respondents reported offering guideline-compatible services. We believe the results of this survey study will inform interventions that target barriers to pediatrician guideline adherence and thereby reduce the incidence of peanut allergy in infants.

## Notes

Supplement. **eAppendix.** Pediatrician Survey Questionnaire **eFigure.** Flow Diagram of Study Participants’ Disposition **eTable 1.** Sensitivity Analysis of Primary Outcome, No. (%) [95% CI] **eTable 2.** Selected Outcomes by Practice Characteristics, No. (%) [95% CI]
