# Supplementary material for: Assessment of Pediatrician Awareness and Implementation of the Addendum Guidelines for the Prevention of Peanut Allergy in the United States
Source: JAMA Netw Open. 2020 Jul 15;3(7):e2010511. doi: 10.1001/jamanetworkopen.2020.10511 (PMC7364336; doi:10.1001/jamanetworkopen.2020.10511)
Supplement: Supplement. — eAppendix. Pediatrician Survey Questionnaire eFigure. Flow Diagram of Study Participants’ Disposition eTable 1. Sensitivity Analysis of Primary Outcome, No. (%) [95% CI] eTable 2. Selected Outcomes by Practice Characteristics, No. (%) [95% CI] [file jamanetwopen-3-e2010511-s001.pdf]

Supplementary Online Content

Gupta RS, Bilaver LA, Johnson JL, et al. Assessment of pediatrician awareness and implementation of the Addendum Guidelines for the Prevention of Peanut Allergy in the United States. *JAMA Netw Open*. 2020;3(7):e2010511. doi:10.1001/jamanetworkopen.2020.10511

eAppendix. Pediatrician Survey Questionnaire

eFigure. Flow Diagram of Study Participants' Disposition

eTable 1. Sensitivity Analysis of Primary Outcome, No. (%) [95% CI]

eTable 2. Selected Outcomes by Practice Characteristics, No. (%) [95% CI]

This supplementary material has been provided by the authors to give readers additional information about their work.

©2020 Gupta RS et al. *JAMA Network Open*

eAppendix. Pediatrician Survey Questionnaire

PEDIATRICIAN SURVEY QUESTIONNAIRE

The *Addendum Guidelines for the Prevention of Peanut Allergy in the United States: Report of the NIAID-Sponsored Expert Panel,* published in 2017*,* contains 3 guidelines based on an infant's risk of developing peanut allergy. Throughout this survey, these new guidelines are referred to as the 2017 Guidelines.

1. What is your primary medical specialty?
    a. Pediatrics
    b. Allergy and Immunology (End survey, provide message)
    c. Family Medicine (End survey, provide message)
    d. Internal Medicine (End survey, provide message)
    e. Other (End survey, provide message)

    (Message: Provide a thank-you message stating that the survey is targeting pediatricians and that he/she will receive the compensation stated in the invitation letter)
2. Do you provide general pediatric care to infants ≤12 months of age?
    a. Yes
    b. No (End survey; provide a thank-you message stating that the survey is targeting pediatricians in general practice who provide care to infants and that he/she will receive the compensation stated in the invitation letter)
3. Which of the following peanut-allergy services, if any, do you provide for infants? (Select all that apply)
    a. Advising parents on peanut allergy prevention
    b. Peanut-specific IgE testing
    c. Peanut-specific skin prick testing
    d. In-office, supervised feedings of a peanut-containing food
    e. Graded oral food challenges
    f. Referrals to an allergist
    g. None of the above
4. Indicate your level of agreement with the following statement:

    The early introduction of peanut-containing foods is an effective method for the prevention of peanut allergy.

    a. Strongly agree
    b. Agree
    c. Neither agree nor disagree
    d. Disagree

e. Strongly disagree

5. Prior to this survey, were you aware of the 2017 Guidelines that recommend the early introduction of peanut-containing foods into the diets of infants to prevent peanut allergy?
   a. Yes
   b. No (Go to Q13)

(Q6-Q10 are for respondents who are aware of the 2017 Guidelines)

6. What have been your sources of information on the 2017 Guidelines? (Select all that apply)
   a. Word of mouth from medical colleagues
   b. News stories
   c. Medical journals
   d. Articles or notices from professional organizations
   e. Local, state, national, or international medical meetings
   f. Continuing medical education course
   g. Online tutorial or course
   h. Expert lecture or grand rounds
   i. In-service training within my practice
   j. My residency or fellowship
   k. Advocacy or healthcare organizations
   l. Online social media
   m. Other(s):________________________(Type in responses)

7. Prior to this survey, how familiar were you with the content of the 2017 Guidelines?
   a. Very familiar
   b. Somewhat familiar
   c. Not at all familiar

8. How much of the 2017 report entitled "Addendum Guidelines for the Prevention of Peanut Allergy in the United States: Report of the NIAID-Sponsored Expert Panel" have you read?
   a. I have read the full report
   b. I have read parts of the full report
   c. I have read only a summary of the report
   d. I have NOT read any of the full report or a summary of the report

9. Do you believe you need more education or training on the 2017 Guidelines?
   a. Yes
   b. No

©2020 Gupta RS et al. *JAMA Network Open*

10. Which statement best describes your use of the 2017 Guidelines in your practice?

   a. I am NOT using the 2017 Guidelines in my practice
   b. I am using parts of the 2017 Guidelines but not all of it (Go to Q12)
   c. I am using the 2017 Guidelines as published and rarely deviate from any part (Go to Q12)

(Q11 is for respondents who are NOT using the 2017 Guidelines: Q10a)

11. Which of the following are reasons you are NOT using the 2017 Guidelines in your practice? (Select all that apply)

   a. I do not have enough clinic time
   b. The Guidelines are too new
   c. I do not know enough about the Guidelines
   d. The practice that employs me has not adopted the Guidelines
   e. The Guidelines are too difficult to understand and apply
   f. The clinical evidence for the Guidelines seems insufficient
   g. I disagree with part or all of the Guidelines
   h. I do not provide some of the peanut-allergy services required by the Guidelines
   i. I do not have access to an allergist for referrals
   j. Insurance coverage or reimbursement is insufficient
   k. I do not believe parents will be interested
   l. I believe parents will be concerned about allergic reactions
   m. I believe parents will be concerned about blood draws
   n. I am concerned about the potential for allergic reactions
   o. I am concerned about legal liability
   p. Other(s):________________________(Type in responses)

   (Respondents of Q11 go to Q13)

(Q12-Q18 are for respondents who are using the 2017 Guidelines: Q10b or Q10c)

12. Among all infants you see at well-child visits, how many receive any aspect of the 2017 Guidelines, such as the assessment of peanut-allergy risk factors, peanut-specific IgE antibody testing, referrals to an allergist, or recommendations about the early introduction of peanut-containing food?

   a. All
   b. Most
   c. Some
   d. None

©2020 Gupta RS et al. *JAMA Network Open*

The next 3 questions ask what you would typically do next for an infant aged 6 months with respect to peanut allergy prevention after assessing the infant for eczema and food allergies.

13. For an infant aged 6 months who does NOT have eczema or any food allergies, what would you typically do next with respect to peanut allergy prevention?

    (Select only one)

    a. Order a peanut-specific IgE test
    b. Conduct peanut-specific skin prick testing in my office
    c. Refer to an allergist for consultation and testing
    d. Recommend avoidance of peanut-containing foods
    e. Offer an in-office feeding of a peanut-containing food
    f. Recommend the introduction of peanut-containing food, in accordance with family preferences and cultural practices
    g. Other:________________________(Type in response)
    h. I would not take any additional steps with respect to peanut allergy prevention

14. For an infant aged 6 months who has mild-to-moderate eczema, what would you typically do next with respect to peanut allergy prevention?

    (Select only one)

    a. Order a peanut-specific IgE test
    b. Conduct peanut-specific skin prick testing in my office
    c. Refer to an allergist for consultation and testing
    d. Recommend avoidance of peanut-containing foods
    e. Offer an in-office feeding of a peanut-containing food
    f. Recommend the introduction of peanut-containing food
    g. Other:________________________(Type in response)
    h. I would not take any additional steps with respect to peanut allergy prevention

15. For an infant aged 6 months who has severe eczema and/or egg allergy, what would you typically do next with respect to peanut allergy prevention?

    (Select only one)

    a. Order a peanut-specific IgE test
    b. Conduct peanut-specific skin prick testing in my office
    c. Refer to an allergist for consultation and testing
    d. Recommend avoidance of peanut-containing foods
    e. Offer an in-office feeding of a peanut-containing food

f. Recommend the introduction of peanut-containing food

g. Other:______________________(Type in response)

h. I would not take any additional steps with respect to peanut allergy prevention

(Respondents to Q15 and Q5b go to Q19; Respondents to Q15 and Q11 go to Q19)

16. Which of the following items have been a barrier or concern for you in using the 2017 Guidelines? (Select all that apply)

a. Lack of clinic time

b. The newness of the Guidelines

c. Understanding and correctly applying the Guidelines

d. I disagree with part or all of the Guidelines

e. Conducting peanut-specific IgE antibody testing

f. Conducting an in-office, supervised feeding of peanut

g. Access to an allergist for referrals

h. Insufficient insurance coverage or reimbursement

i. Parents who are not interested

j. Parental concerns about allergic reactions

k. Parental concerns about blood draws

l. My concerns about allergic reactions

m. Legal liability

n. Other(s):______________________(Type in responses)

o. I have not had any barriers or concerns

17. Which of the following office materials would you like to have available for parents? (Select all that apply)

a. A waiting room poster about peanut allergy prevention

b. A paper or electronic handout explaining the 2017 Guidelines

c. A paper or electronic handout that provides answers to Frequently-Asked-Questions

d. A paper or electronic handout on the feeding of peanut-containing foods at home

e. Other(s):______________________(Type in responses)

f. I am not interested in any office materials for parents

18. To assist you in using the 2017 Guidelines, which of the following practice aids would be helpful to you? (Select all that apply)

©2020 Gupta RS et al. *JAMA Network Open*

a. An online tutorial on 2017 Guidelines implementation
b. Prompts in the electronic medical health record
c. A printed or electronic handout to guide clinical assessments and recommendations
d. A printed or electronic script for explaining the 2017 Guidelines to parents
e. A printed or electronic handout to guide an in-office supervised feeding
f. Other(s):________________________(Type in responses)
g. I am not interested in any practice aids

(Q19-Q29 are for all respondents)

19. What is your gender?
    a. Female
    b. Male
    c. Other:________________________(Type in response)
20. What is your ethnicity?
    a. Hispanic or Latino
    b. Not Hispanic or Latino
21. What is your race?
    (Select all that apply)
    a. American Indian or Alaska Native
    b. Asian
    c. Black or African American
    d. Native Hawaiian or Other Pacific Islander
    e. White
    f. Other:________________________(Type in response)
22. In what year did you graduate from medical school?
    a. _____YYYY
23. Are you a resident or fellow in training?
    a. Yes
    b. No
24. In a typical week, how many hours do you spend in pediatric patient care?
    a. __ Hours per week
25. What is the location of your pediatric practice? (If more than one pediatric practice, the practice at which you spend the most time.)
    a. Urban

b. Suburban

c. Rural

26. Is your pediatric practice owned by or affiliated with a university or other academic institution? (If more than one pediatric practice, the practice at which you spend the most time.)

a. Yes

b. No

27. What type is your pediatric practice? (If more than one pediatric practice, the practice at which you spend the most time.)

a. Private practice: solo practice

b. Private practice: group practice

c. Academic medical center practice or clinic

d. Hospital practice or clinic

e. Community clinic or community health center

f. Managed care organization/HMO

g. Military or U.S. government

h. Other:_______________________(Type in response)

28. In what state is your pediatric practice located? (If more than one pediatric practice, the practice at which you spend the most time.)

a. ___________ (Select from the drop down menu)

29. Approximately what percentage of your pediatric patients are on Medicaid?

a. 0-25%
b. 26-50%
c. 51-75%
d. 76-100%

eFigure. Flow Diagram of Study Participants' Disposition

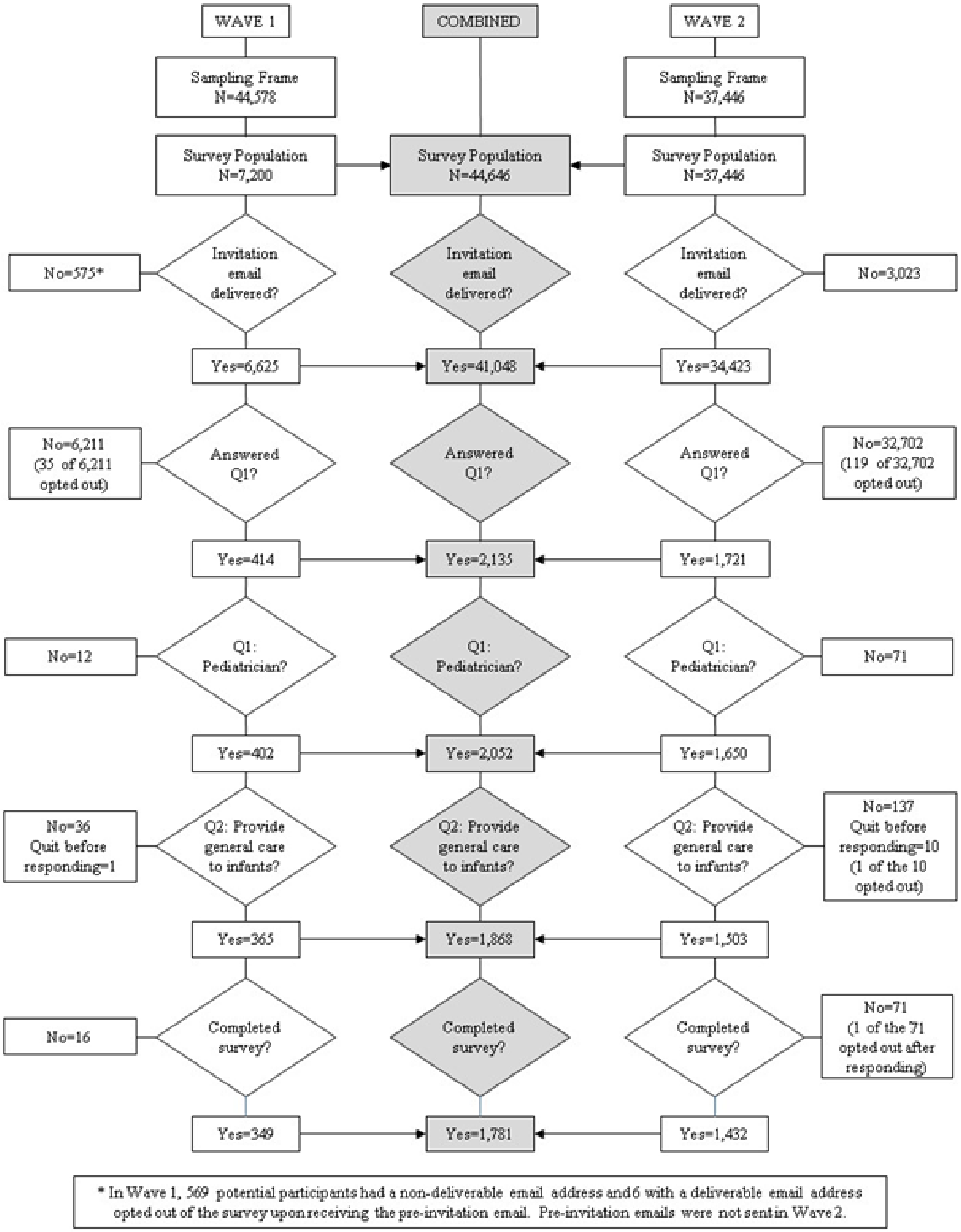

Note: Q1= What is your primary medical specialty? Q2= Do you provide general pediatric care to infants ≤12 months of age?

©2020 Gupta RS et al. *JAMA Network Open*

eTable 1. Sensitivity Analysis of Primary Outcome, No. (%) [95% CI]

	**Percentage of Patients by Wave**		**Percentage of Patients by Incentive Level ($10 vs $50)**	
	**Wave 1**	**Wave 2**	**$10 Incentive**	**$50 Incentive**
**Pediatricians Awareness of Guidelines Prior to Survey**				
Aware	335 (92.5) [89.3 - 95.0]	1390 (93.6) [92.2 - 94.8]	1589 (93.7) [92.4 - 94.8]	136 (90.1) [84.2 - 94.3]
Not Aware	27 (7.5) [5.0 - 10.7]	95 (6.4) [5.2 - 7.8]	107 (6.3) [5.2 - 7.6]	15 (9.9) [5.7 - 15.9]
**Pediatricians Implementing Guidelines**				
Not Implementing	34 (10.1) [7.1 - 13.9]	82 (5.9) [4.7 - 7.3]	100 (6.3) [5.2 - 7.6]	16 (11.8) [6.9 - 18.4]
Partially Implementing	207 (61.8) [56.4 - 67.0]	898 (64.9) [62.4 - 67.5]	1021 (64.5) [62.1 - 66.9]	84 (61.8) [53.1 - 70.0]
Fully Implementing	94 (28.1) [23.3 - 33.2]	403 (29.1) [26.8 - 31.6]	461 (29.1) [26.9 - 31.5]	36 (26.5) [19.3 - 34.7]
Note: Percentages may not add to 100 due to rounding.				

©2020 Gupta RS et al. *JAMA Network Open*

eTable 2. Selected Outcomes by Practice Characteristics, No. (%) [95% CI]

	Percentage of Patients with Medicaid					Pediatric Practice Location			
	**0-25%**	**26-50%**	**51-75%**	**76-100%**	**P-value**	**Rural**	**Suburban**	**Urban**	**P-Value**
**Pediatricians Implementing Guidelines**									
Not Implementing	42 (6.3) [4.6 - 8.5]	27 (6.0) [4.0 - 8.6]	23 (7.0) [4.5 - 10.4]	24 (10.3) [6.7 - 14.9]	0.03	15 (8.2) [4.7 - 13.2]	59 (6.4) [4.9 - 8.2]	42 (7.4) [5.4 - 9.9]	0.03
Partially Implementing	417 (63.0) [59.2 - 66.7]	286 (63.8) [59.2 - 68.3]	222 (67.9) [62.5 - 72.9]	146 (62.7) [56.1 - 68.9]		119 (65.4) [58.0 - 72.3]	572 (61.8) [58.6 - 65.0]	382 (67.3) [63.2 - 71.1]	
Fully Implementing	203 (30.7) [27.2 - 34.3]	135 (30.1) [25.9 - 34.6]	82 (25.1) [20.5 - 30.1]	63 (27.0) [21.4 - 33.2]		48 (26.4) [20.1 - 33.4]	294 (31.8) [28.8 - 34.9]	144 (25.4) [21.8 - 29.1]	
**Pediatricians Reporting a Need for Training**									
Yes	415 (62.7) [58.9 - 66.4]	313 (69.9) [65.4 - 74.1]	242 (74.0) [68.9 - 78.7]	177 (76.0) [70.0 - 81.3]	<0.001	124 (68.1) [60.8 - 74.8]	612 (66.2) [63.0 - 69.2]	414 (72.9) [69.0 - 76.5]	0.02
No	247 (37.3) [33.6 - 41.1]	135 (30.1) [25.9 - 34.6]	85 (26.0) [21.3 - 31.1]	56 (24.0) [18.7 - 30.0]		58 (31.9) [25.2 - 39.2]	313 (33.8) [30.8 - 37.0]	154 (27.1) [23.5 - 31.0]	
**Pediatricians Reporting Barriers to Implementation (Among Partial or Full Guideline Implementation)**									
Access to Allergists for Referrals	36 (5.8) [4.1 - 7.9]	46 (10.9) [8.1 - 14.3]	31 (10.2) [7.0 - 14.2]	33 (15.8) [11.1 - 21.5]	<0.001	28 (16.8) [11.4 - 23.3]	61 (7.0) [5.4 - 9.0]	57 (10.8) [8.3 - 13.8]	<0.001
Insufficient insurance coverage or reimbursement	36 (5.8) [4.1 - 7.9]	41 (9.7) [7.1 - 13.0]	28 (9.2) [6.2 - 13.0]	24 (11.5) [7.5 - 16.6]	0.006	21 (12.6) [8.0 - 18.6]	66 (7.6) [5.9 - 9.6]	43 (8.2) [6.0 - 10.9]	0.1
Lack of clinic time	153 (24.7) [21.3 - 28.3]	117 (27.8) [23.6 - 32.3]	92 (30.3) [25.1 - 35.8]	84 (40.2) [33.5 - 47.2]	<0.001	52 (31.1) [24.2 - 38.8]	228 (26.3) [23.4 - 29.4]	167 (31.7) [27.8 - 35.9]	0.07
Conducting an in-office - supervised feeding of peanut	185 (29.8) [26.3 - 33.6]	121 (28.7) [24.5 - 33.3]	112 (36.8) [31.4 - 42.5]	82 (39.2) [32.6 - 46.2]	0.003	67 (40.1) [32.6 - 48.0]	278 (32.1) [29.0 - 35.3]	159 (30.2) [26.3 - 34.4]	0.06

Note: Percentages may not add to 100 due to rounding. P-values are from χ^2 tests of association.

©2020 Gupta RS et al. *JAMA Network Open*
